# A Comparative Sociology of Gypsy Traveller Health in the UK

**DOI:** 10.3390/ijerph16030379

**Published:** 2019-01-29

**Authors:** Miranda Millan, David Smith

**Affiliations:** 1 Faculty of Education and Health, University of Greenwich, London SE9 2UG, UK; 2 DAVID SMITH Reader in Social Policy, Faculty of Health, Education, Medicine and Social Care, Anglia Ruskin University, Chelmsford, Essex CM1 1SQ, UK

**Keywords:** Romany Gypsy, Irish Traveller, comparative sociology, social determinants of health, accommodation

## Abstract

This paper presents findings from a series of health-related studies undertaken between 2012 and 2017 with Romany Gypsies and Irish Travellers living in different locations and in various forms of accommodation in southern England. These set out to develop a sociological understanding of the factors impacting on the health and wellbeing of members of those communities and to consider the extent health status is shaped by ethno-cultural and/or socioeconomic factors, and the interplay and direction of causal processes between them. The relative influences of cultural and structural factors in generating health inequalities have important implications for engaging marginalised populations in health services and preventative programmes. This paper will present survey and qualitative data on Gypsies’ and Travellers’ health beliefs and practices to understand how those beliefs and practices have developed in different social contexts as responses to deeper social mechanisms, and share commonalities with other marginalised and excluded social groups. In policy terms this indicates the need for health interventions that are applied proportionate to the level of disadvantage experienced thus ensuring equality and fairness while accounting for diversity and difference.

## 1. Introduction

Romany Gypsies are generally considered those indigenous to England (also known as English Gypsies) while Irish Travellers generally refers to those originally indigenous to Ireland. Whilst these groups share some similarities in terms of lifestyle, economic, family and communal structures and norms and values they are distinctly different ethnic minorities (For an overview of the contemporary situation of Gypsies and Travellers in the UK, please refer to House of Commons Briefing Paper 08083 [1]). The Department for Communities and Local Government (DCLG) defines Gypsies and Travellers as:

“Persons of nomadic habit of life whatever their race or origin, including such persons who on grounds only of their own or their family’s or dependants’ educational or health needs or old age have ceased to travel temporarily, but excluding members of an organised group of travelling showpeople or circus people travelling together as such.”

[2]

While Gypsies and Travellers are often regarded as having the poorest health and lowest life expectancy in the UK such statements have been accepted rather uncritically, and two key barriers to a fuller understanding of the health of these communities remain unaddressed. First is the tendency to make generalisations concerning aggregate health status, inferred from relatively small samples of the population. Such an approach cannot—and generally does not attempt to—identify any patterned commonalities and differences in health status, beliefs, practices and perceptions of health services between different communities living in different geographic locales. Furthermore, this approach is unable to determine the impact on health practices and health-seeking behaviour of supply side factors such as the provision and quality of health services or accessibility of locally available services, which vary widely in different UK regions [3]. Likewise factors that impact on health and are related to socioeconomic status; quality and type of accommodation; access to community and communal support structures, and levels of integration and acceptance or of exclusion and hostility within different localities are rarely accounted for, despite their important impacts on both mental and physical health. The second barrier is the tendency to explain inequalities largely through the prism of ethnicity. This can lead to the assumption, found in much of the health literature as well as the views of many professionals who work with Gypsies and Travellers, of their innate ‘difference’ regarding attitudes and practices concerning health [4,5]. This focus on difference overlooks the significant parallels and similarities in health-related attitudes and practices shared with other sections of the population, while the alleged insular, ‘hard to reach’ nature of Gypsy and Traveller communities is frequently deployed to justify the minimal progress in addressing their health issues. A satisfactory explanation for health inequalities should be multi-factorial and consider interactive effects as well as the expressed views and experiences of community members. A failure to consider these factors can result in the maintenance of stereotypical beliefs and misguided priorities among some health and other service providers regarding what Gypsies and Travellers ‘need’ and a profound disconnect between professional discourses and the lived experiences of Gypsies and Travellers [6]. 

This paper will present findings from a PhD comparative sociological study into the relationship between accommodation, health and wellbeing in two case study areas conducted by the lead author. It will also refer to studies led by the second author, which explored the factors impacting on Gypsy and Travellers’ parental decision-making surrounding childhood immunisation; attitudes, beliefs and practices regarding cancer and their relationship to health-seeking behaviour and the remedial actions that individuals undertake to rectify perceived ill health [7]. While much of the existing literature and evidence relating to Gypsy/Traveller health in the UK has been epidemiological in nature, these studies set out to develop a sociological understanding of the factors impacting on the health and wellbeing of members of those communities. This entails a consideration of the relatively neglected issue of how inter and intra-community patterned differences in health relate to wider social and economic inequalities and conditions.

### Survey Data and Gypsy Traveller Health

Gaining an understanding of the general health status of Gypsies and Travellers in the UK is hampered by the lack of systematic large-scale data or routine monitoring of these populations by health authorities. Despite improved ethnic monitoring being a key priority in the Government’s strategy to reduce ethnic health inequalities, Gypsies, Roma and Travellers are not included in the National Health Services’ (NHS) ethnic monitoring codes meaning that no national level health data exists. Of 125 NHS Primary Care Trusts (PCTs) only 20 (16%) had—or were intending to—introduce health monitoring procedures for Gypsies Roma and Travellers and half of those (10/125) had only conducted partial monitoring [8] (p. 3). The largest health survey to date is Parry et al.’s [9] epidemiological study using standard health measures of 293 Gypsies and Travellers in five locations undertaken for the Department of Health’s ‘Inequalities in Health Research Initiative’. The study sample included matched samples as a comparative group with Gypsies and Travellers of both genders across four age groups (16–25, 26–45, 46–65 and 65+) who were located primarily through local health visitors or other service providers. Parry’s findings confirmed that Gypsies and Travellers experience significantly poorer health at all stages of life and more self-reported ill health than the comparator groups, with findings pointing to the existence of an inverse relationship between health need and use of health services. While a host of smaller scale qualitative localised studies confirm that poor health throughout the life course and premature death is an all too common aspect of Gypsy and Traveller experience, the possibility that survey and other findings are—at least in part—an artefact e.g., a result of the data collection and sampling methods used, is rarely considered. Survey samples of Gypsies and Travellers tend to be drawn from the more ‘visible’ members—those living on sites, by the roadside and those who are known to statutory service providers—often the poorer and more marginalised sections of those communities and those with the poorest health [10]. The diversity of the population is not reflected using this sampling approach. Those with greater economic resources; those who are less visible for example because they live in conventional housing or who do not identify with the ethnic ‘community’, along with those not in contact with service providers or community organisations tend to be under-represented in research findings. Results therefore, can present a skewed image of collective health that lend themselves well to ‘cultural deprivation’ type explanations rooted in lifestyle, ignorance and misguided health beliefs, and which downplays the role of social structural factors and the ethnic and power relations through which inequalities are generated and sustained [11]. 

In essence many of the challenges facing policy makers aiming to improve health services and outcomes for Gypsies and Travellers are not too dissimilar to those of other poor and marginalised groups, e.g., material factors such as poverty, diet, lifestyle, poor accommodation and working conditions all of which increase multiple risk exposure which in turn, increases physical and mental health risks [12]. However, while socioeconomic factors partly explain health inequalities, adjusting for social class reduces ethnic health inequalities but does not eliminate them [13]. Different minority groups have diverse experiences in their relations with the majority society, with respect to experiences of discrimination and social exclusion or conversely, of social acceptance and assimilation [14]. This profoundly shapes their societal experiences and more specifically, their experiences in health care settings and propensity to access such services, with a body of research demonstrating that Gypsies and Travellers fare worse than most other groups across a range of domains including use of health and other services [15]. Such findings suggest the need for health interventions that are applied with a scale and intensity proportionate to the level of disadvantage experienced, meaning that improving the overall health of Gypsies and Travellers may require a more concentrated and intensive approach due to the multifactorial disadvantages that a disproportionate number of those community members experience [16]. 

## 2. Materials and Methods

### Comparative Case Study: West London and South Buckinghamshire

Localised patterns of poverty, work, and demographic factors such as age structure and gender are important when considering the impacts of location on health both within and between different ethnic groups [17] (p. 100). The paucity of comparative health studies and investigation of intra-group differences in relation to Gypsy and Traveller health in the academic and policy literature, means these areas of enquiry are largely unexplored [9] (p. 6). The following mixed methods study conducted as part of a doctoral thesis is one of few comparative studies of Gypsy and Traveller health in the UK. Findings demonstrate the distinctly localised patterning of health and wellbeing between two communities in two different localities and allows for the wider social determinants of health to be considered when understanding those patterns. The primary research undertaken was a cross- sectional, sequential exploratory, mixed methods case study that sought firstly to explore the correlations between accommodation, planning situations (for official permission to develop residential caravan sites) and the health and wellbeing of Romany Gypsy families in South Buckinghamshire and Irish Traveller families in Ealing, West London; and secondly, to uncover the general mechanisms that influence health and the specific forms it takes in terms of health and wellbeing in the two localities. 

Qualitative field research was carried out between October 2015 and September 2017 in the two areas. Participants were gathered using non- probability purposive sequential sampling in the form of maximum variation referral sampling, sometimes also called snowball sampling. This consisted of participant observation and in-depth interviews with 19 Irish Travellers and seven professionals who worked with Gypsies and Travellers in Ealing, and 13 Romany Gypsies and six professionals in South Buckinghamshire (Bucks). Interview questions were based on the literature review findings and had three main sections covering accommodation; health; and any intersection between the two. All interviews were recorded and then transcribed. After the main themes and issues from the qualitative phase had been analysed by the lead author using thematic analysis the findings were used to generate a survey to test for scale, scope and prevalence of the themes within the wider Gypsy Traveller populations in the case study areas. Quantitative data was collected between February and March 2018 using a survey completed by 109 Gypsy and Traveller residents in the two case study areas of whom 57 (52%) reside in the London Borough of Ealing and 52 (48%) reside in South Bucks District. The questionnaire participants were self-selected using a convenience non- probability voluntary sampling method, which is generally considered an acceptable approach for a pilot questionnaire such as this. Participants were invited to complete questionnaires online, in person or over the telephone. Links to the questionnaire were disseminated through interview participants (Gypsy Traveller and professionals) as well as through adverts placed on relevant online message boards and social media. A member of the Irish Traveller community assisted to complete paper questionnaires, by which non- literate participants, those lacking Internet access or those lacking computer skills could be included. There were no incentives offered for taking part in either stage of the research, apart from the opportunity to share their thoughts and opinions. Whilst the lead researcher knew the majority of qualitative and quantitative participants from Ealing Borough, the researcher did not previously know the majority of those from South Bucks.

Informed consent was obtained from all qualitative and quantitative participants. Interview subjects were asked to sign a consent form, which was read aloud to them, in order to indicate their willingness to take part in the research. Questionnaire respondents gave their informed consent online by ticking a box on the landing page of the questionnaire host website. Those completing questionnaires in person were read the research consent statement and asked to indicate their consent by ticking the box, and telephone respondents were asked to consent verbally after hearing the consent statement. 

## 3. Results

### 3.1. Demographic, Household and Socioeconomic Characteristics 

The two case study communities demonstrated distinct patterns of residence with 98% of respondents in South Bucks identifying as Romany Gypsies while in Ealing 89% of respondents identified as Irish Travellers and 9% as Romany Gypsies. The majority of respondents in both case study areas were women although there was notable variation between Ealing (79% female) and South Bucks (54%). The largest age cohort of survey respondents were aged between 21 and 30: in Ealing 39% of respondents were in this age range and 37% in South Bucks.

The following statistical results are drawn from the quantitative survey undertaken and are illustrated where appropriate with excerpts from the qualitative interviews. Interviews with professionals in both case study areas broadly mirrored topics and perceptions covered in interviews with Gypsy Travellers. However significant areas of divergence are highlighted as part of the discussion which follows. In terms of accommodation type, household structure, income and receipt of state welfare benefits there were marked differences between the two areas. The majority of the Ealing sample were resident in conventional housing (73%) with 13% living on caravan sites and 14% homeless at the time of the survey. This compared to South Bucks where 48% lived in housing and 52% lived on sites. Variation in accommodation type reflects the different nature of the respective locales (peri-urban and urban) as South Bucks has considerably more sites than Ealing, more open space and reasonably priced land therefore making it more receptive to the development of private sites, for which there is a precedence, as well as historically public sites (although these have now been sold and are in private hands). In contrast Ealing, being an outer London Borough, suffers from the inadequate social housing, inflated private rental prices and high cost of land endemic to the entire capital. As such poor conditions in housing and the prevalence of temporary accommodation was mentioned only by interviewees in Ealing and not by those in South Bucks. 

The two samples also differed significantly in terms of household composition and socioeconomic status. Households in South Bucks were more likely to have a wife/husband/partner living in the household (57%) than those in Ealing where only 38% reported this. Over half (51%) of households in Ealing lived with children under 16 and were more likely to have come from large families (3+ children) themselves whereas in South Bucks just 32% of households lived with their children and tended to live in smaller family units. UK annual median disposal income in 2017 was £27,300 and the majority of respondents in both case study areas fall below this threshold [18]. Households with incomes less than 60% of median earnings—approximately £16,380—are below the official UK poverty line and on this estimate a significant number of households in both localities who took part in this survey are in poverty, though with notable differences. Ealing residents were at considerably higher risk of poverty with 89% reporting a household income of less than £20,000 and none reporting an income in excess of £40,000. For South Bucks the distribution is spread more equally with 44% reporting an annual household income of under £20,000 and 20% earning less than £30,000. Conversely 22% of South Bucks respondents had incomes in excess of £40,000 and 8% earned over £50,000 per annum. 

The determinants of poverty also varied between the two areas with low wages being a more significant driver of low household income in South Bucks than in Ealing, where unemployment and economic inactivity was more significant. Whilst approximately one third (31%) of survey respondents in South Bucks were in receipt of some form of welfare benefit—roughly equal to the UK average—three quarters (75%) of the Ealing respondents were in receipt of state benefits. Very few of the South Bucks survey sample received out of work benefits and most of those claiming benefits were claiming tax credits, an in-work wage supplement. In Ealing survey respondents reported higher levels of out of work benefits such as ‘Income Support’ and ‘Employment and Support Allowance’ compared to South Bucks. It is worth noting however that what people report to surveys about their income and household compositions should not be taken at face value, particularly when these are implicated in eligibility for welfare benefits [19].

Divergence between the two areas is largely due to the geographical and socio-economic differences, for example a lack of caravan sites in Ealing and a general housing shortage that effects the entire capital city that for the Irish Travellers in Ealing, manifests itself in more poor quality housing and a prevalence of temporary accommodation, social and privately rented housing when compared to South Bucks. Importantly these differences were also reflected in significantly differing levels of satisfaction with their accommodation reported to the survey between the two locales, and in the overall health and wellbeing of the two survey samples (discussed below). The data indicates that the same issues are experienced in both, but among the Ealing sample the issues are intensified and concentrated.

### 3.2. Gypsy and Traveller Households’ Self-Reported Health

The Gypsies and Travellers sampled in both localities had a poorer health profile than the UK average. To record instances of self-reported physical and mental health among survey respondents two separate questions; whether the respondent, or anyone in their household had in the past two years suffered any physical; or any mental health problems. Respondents were also given the option to give free- text responses to elaborate.

Approximately half of all Ealing survey respondents said that they or someone in their household has a disability compared to approximately 25% of those in South Bucks, which are both higher than the UK average of 19% [20]. Ealing residents were almost twice as likely as those in South Bucks to report physical health problems in their household in the preceding two years (67% compared to 34%). The Ealing sample were also more than twice as likely compared to those in South Bucks to report mental health problems in their household in the preceding two years (71% compared to 34%). This compares to 17% of UK adults over 16 reporting a mental health problem in the week prior to interview, though 43% of UK adults have had a mental health problem at some point in their lives [21]. It was clear during the qualitative interviews that mental health and suicide are serious concerns for both communities. Eight interviewees (three from South Bucks and five from Ealing) spoke about their personal experiences of losing family members to suicide, a phenomenon particularly common among young Gypsy and Traveller men (but also increasingly among women as well). Irish Travellers for example, have a suicide rate three times that of the general population [15]. Interviewees identified suicide risk factors to be prison, moving from a site and/or living ‘on the road’ into housing for the first time, and poor employment prospects (again, impacting on men in particular). Interviewees in both locations highlighted the prevalence of depression and anxiety among Gypsy and Traveller communities of both sexes. Every interviewee in Ealing mentioned depression or mental health problems at some point during their interview, whereas in South Bucks around half mentioned these issues. 74% of survey respondents in South Bucks and almost all Ealing respondents (98%) feel that members of their community suffer from higher levels of stress than average. When asked the causes of stress survey respondents in both areas mentioned ‘lifestyle’, which encompassed racism and discrimination; shortages of appropriate accommodation and site provision and a lack of understanding, combined with the prevalence of negative stereotypes about their culture.

A frequent theme during the interviews in both areas was the negative health impacts in older age of having lived nomadically when they were younger. All of those interviewed had previously lived in caravans on the road, and many (especially among the Ealing sample) were born on the road. Many of the older interviewees felt that living on the road had meant missing out on essential services such as healthcare, education and basic facilities, and that this in conjunction with often harsh outdoor living conditions and frequent stops on unsanitary and polluted land (by motorways, on disused industrial sites and wasteland etc.) has had a cumulative negative impact on their health. Despite the significant hardships and privations that living on the road entailed, there is a palpable nostalgia when interviewees discuss their memories and experiences of living on the road. In addition, interviewees in South Bucks spoke about the health impact of traditional trades such as working on farms and in scrap metal. A male respondent in South Bucks discussed how restricted work options channelled Gypsies and Travellers into the most physically demanding types of work, which in many cases has had long-term health impacts.

We’ve got a lot of field workers and my wife worked in different field work and things, and yes it’s made a big impact, I believe that, because there wasn’t any other jobs, that was classed suitable. It was either scrap metal, going out on the scrap or it was field work… They were some of the main, you know, farm worker, were some of the main things because they were the only people that would employ us… and of course of a morning when they were in fields at 4 o’clock in the morning cutting, I never done it you see, now their hands have gone, yeah of course it affects their health yeah, most definitely.

This would only tend to affect those over the age of 40 given the gradual decline of seasonal agricultural and other traditional types of work for Gypsies and Travellers in the past 50 years. Indeed, given the reduced demand for the goods and services that were previously supplied by this nomadic and seasonal workforce and fewer job opportunities for low and unskilled workers generally, a pressing concern of Gypsy and Traveller communities is improving the poor educational outcomes of their youth [22].

### 3.3. Accommodation and Health

Previous studies have highlighted that conventional housing can be a double-edged sword for many Gypsies and Travellers. While being settled allows greater access to health, education and other services this was in many cases offset by the negative impacts on mental health such as isolation, claustrophobia and depression [23,24]. This is supported by the research findings in Ealing and South Bucks, which finds a correlation between self- reported health and wellbeing (as discussed above) and type of accommodation, especially in Ealing where there are higher numbers in bricks and mortar accommodation. As discussed around three quarters of the Ealing survey respondents lived in housing compared to just under half of those in South Bucks. Nearly three quarters of Ealing residents (72%) feel that accommodation impacts on the health and wellbeing of their household compared to South Bucks where this falls to just under half of respondents (48%). One of the Ealing interviewees made a connection between the increasing numbers of housed Travellers and the deterioration of traditional community and extended family structures, summarising the impact of housing as follows:

Yeah for health wise, it’s good to be in a permanent place, but I think for health, if you’re in a house you don’t want to be, where you want to be on the road. I don’t mean out on the road, I mean in a site, we were reared in caravans so being in a caravan we’re more happy, but when we’re in a house, like I said, it’s like we’re imprisoned. So that’s the high rate of depression and suicide among Travellers now, since they’re forced into houses and stuff.

The South Bucks survey sample residing in housing are more likely to own their own home (46%) while those in Ealing are more likely to live in social housing (63%). Social housing in the UK has been increasingly transformed from affordable ‘general needs’ housing into welfare housing for the poorest, economically inactive and most deprived sections of the population in recent decades [25]. Differences in housing tenure between the Ealing and South Bucks survey samples reflect the differences in household income and the fact that the Ealing sample are generally poorer than those in South Bucks. Likewise, in South Bucks the majority of respondents living on a caravan site live on a private site indicating greater economic resources to purchase and develop land, with a handful living on a site that was until recently publicly owned. Interviewees resident on the public caravan site in Ealing reported considerable concerns about the location of their site, the state of repair and the impact of environmental factors on their health and wellbeing. By contrast no such concerns were raised by residents interviewed on several sites in South Bucks that until recently were publicly owned, indicating variation in the quality of sites between the two locales, with implications for the health and wellbeing of residents [26]. Eighty-eight percent of survey respondents in South Bucks and 89% of the Ealing respondents reported the planning process for private sites and difficulties gaining planning permission to be an extremely stressful process. One interviewee from South Bucks noted that:

Planning is a very long process and not always straight forward. It can cost thousands to buy land and then more money to apply for planning. Many occasions this results in land not being passed and still the families are homeless. The council don’t provide sites for Gypsy people. Many of my community struggle to settle in one place due to planning. This causes excess stress for all involved, including children.

Most qualitative and quantitative respondents in both areas expressed a preference for living on a site and differences in accommodation type across the two areas are reflected in contrasting levels of satisfaction with their current accommodation. In South Bucks 80% of survey respondents would choose to remain living in their current accommodation given the choice whereas this figure falls to 47% in Ealing. Type of accommodation also impacts on the ability to receive assistance and support from their community since living on a site facilitates an extended family and communal lifestyle unlike housing which is largely designed for a nuclear family structure. The majority of survey respondents in Ealing (59%) do not feel close to their community in their current accommodation and 74% feel that their accommodation makes it difficult to receive community support. Conversely 78% of respondents in South Bucks feel close to their community in their current accommodation and 58% do not feel that their accommodation hinders them from receiving community support. 

The majority of survey respondents in both areas felt that the health and wellbeing of their communities is worse than that of the settled population but as with previous responses, a trend among South Bucks respondents for a more positive outlook was apparent with most stating that the health and wellbeing of their community is improving (62%), a minority feeling it is staying the same (34%) and only a fraction feeling that it is getting worse (4%). In Ealing the opposite view was expressed, with more feeling that the health of their community is getting worse (43%), but still over half of respondents feeling it is either ‘staying the same’ or ‘getting better’ (57%). 

### 3.4. Access to Health Services by Location

Barriers accessing health services including discrimination in health care settings have been posited as major factors explaining Gypsies and Travellers’ low access to health services [9,27]. The findings from the survey data and qualitative interviews in the current study diverge from much of the literature in this regard. When asked whether they felt that they had the same unprejudiced access to healthcare services as non-Gypsy Travellers the majority of respondents—96% in South Bucks and 85% in Ealing responded in the affirmative. Similarly, survey data found that the vast majority of respondents in South Bucks (92%) and a majority in Ealing (62%) do not feel discriminated against when accessing healthcare. An interviewee in South Bucks responding to whether he or his family had ever received poor treatment in health care settings replied:

Not at all. We don’t. We personally haven’t received prejudice in that area with any doctor and we’ve lived here a lot of years. A lot of them do know my family and they know that we’re well-mannered people, like I’ve said. How other people are treated, I don’t know, but we’ve been treated with the fullest of respect and probably gone out their way, out their way to give us treatment.

However, a significant minority in Ealing (38%) report feeling discriminated against when accessing healthcare and it is important to note that discrimination accessing statutory services (including healthcare) is reported as widespread and supported by a large body of evidence [27,28,29]. Preconceptions of discrimination may also impact healthcare access, even if no discrimination is subsequently experienced. Examples were given in both case study areas that an anticipation of discrimination is expected from GP surgeries for example. The following interviewee from Ealing explains this:

Yeah definitely, there is a perception there is a fear of social workers, health visitors, doctors, schools, all looking down on you as though you’re not good parents and stuff. And obviously Travellers is good parents, they do the best they can do given the circumstances, but there is that perception there.

Furthermore a few interviewees in Ealing, whilst stating they felt that they had non-prejudicial access to healthcare services, simultaneously reported experiencing discrimination from individual GPs such as not being listened to or serious ailments being treated as minor complaints though such complaints are also common among the wider public [30]. No complaints were made against care given by other services however, with many making positive comments about the care given especially by hospital services and Accident & Emergency (A&E) provision. 

An important caveat to the discussion below is that all the survey sample and all but two (one in South Bucks and one in Ealing) of the interview sample was settled either on sites or in housing at the time the research was undertaken. Those who continue to pursue a nomadic lifestyle (either on the road or within housing stock) do not share the benefits of equal access. In Ealing and South Bucks the sentiment was expressed by numerous interviewees, including the only ‘roadsiders’ interviewed that there are still significant barriers to accessing appropriate healthcare and continuity of care whilst ‘on the road’. It should be noted that NHS initiatives such as walk-in clinics and the 111 telephone service have fairly recently been introduced and were mentioned as useful by one roadsider. Those respondents now residing in housing may be referring to an historic lack of roadside access. 

## 4. Discussion

In both areas most survey respondents felt that their household has the access to healthcare that it requires and that the healthcare their family receives meets their needs. This underlines the need for close exploration of the specific constellation of health service provision, access, attitudes and experiences with regards to healthcare in different locales. Localised perceptions of healthcare among particular communities of Gypsies and Travellers and the reality of their engagement with the healthcare system may be at odds with generalised claims in much of the academic and policy literature concerning low access, reluctance to engage with health services and pervasive discrimination in the healthcare system. A more finely grained and robust comparative analysis would make it possible to identify the variables that make particular populations of Gypsies and Travellers more or less favourable to healthcare in their areas. Graham Scambler [31] (p. 166) highlights the importance of considering how the ethnic patterning of health relates to wider social and economic inequalities, a consideration that has not received the prominence it warrants in discussions of Gypsy and Traveller health in the UK. As a result, discourses that homogenise these populations and propose naively uncritical and undifferentiated accounts of their health predominate. In the two localities reported here social determinants play an important role in explaining variations in the health and wellbeing of the two Gypsy/Traveller communities. Studies have shown how living on unauthorised encampments [24]; on run down overcrowded public sites [26,32] or of being forced into low quality housing isolated from community structures can have damaging outcomes on physical and mental health [9,33]. The Ealing Travellers were largely living in housing where the practical difficulties of adapting to life in bricks and mortar can combine with hostility and conflict with neighbours to exacerbate stress [23]. They were also more likely than the South Bucks participants to have experienced eviction, financial hardship, damp/rot, pest infestation, poor state of repair and problems with neighbours. Notwithstanding the problems inherent in drawing conclusions regarding income and household composition from survey data, they were also significantly poorer on the whole and more likely to be receiving out of work welfare benefits than the South Bucks sample. The latter were more likely to be living on a private site with their family—the preferred option of most Gypsies and Travellers in this study—to have greater access to social support structures and in general, possessed greater economic and material resources. The Gypsies in South Bucks are also more likely to be living with their spouse or partner and less likely to have children under 16 living in the same household. Larger family size is closely associated with poverty: 39% of households with three or more children in the UK are living in poverty compared to 26.5% of households with one or two children in 2015/16 [34]. In Ealing households were more likely to contain children and to have larger families with 45% of those with children reporting three or more compared to 31% in South Bucks. The latter were also a longer established community in the locality having been resident in many cases for several generations, and had integrated into the wider community to a greater degree. By contrast many of the Ealing Travellers had more recently experienced the transition from living on the road to living in housing in a highly urbanised and ethnically diverse environment. 

These structural and historical coordinates set the boundaries within which lives are experienced and intersect with ideological factors such as prejudice and discrimination to produce inequitable health outcomes on one hand, and shared (though not identical) experiences and dispositions towards health and the health system on the other. However, structural factors do not act in a uniform manner but are mediated at the local level by a multitude of intervening factors that combine to produce patterned variations in health and wellbeing within specific populations. Atkin [35] (p. 128) notes that ethnicity denotes more than group membership but is ‘an expression of a person’s negotiation of multiple identities within different social and historical contexts’. It is the interplay between ethnicity and those local contexts that play a pivotal role in shaping health outcomes. Nevertheless, the idea that health inequalities generated at the structural level is attributable to cultural attitudes and practices and can therefore be reduced solely by changing attitudes and behaviour, has been the dominant influence on health promotion strategies. The implications of these approaches for improving the health of Gypsies Travellers and other socially excluded populations are considered below.

The role of cultural and structural factors has important implications for engaging marginalised populations in health services and are based in contending views of causation with the former emphasising the role of collective health beliefs and practices in generating and perpetuating poor health. The latter in attempting to illuminate the ‘causes of causes’ [36] emphasises the social context that generates those shared systems of beliefs, behaviour and experiential patterns meaning that restructuring and reform of the economy and society are necessary preconditions for reducing health inequalities. These competing discourses have important ramifications when they underpin policy interventions or are deployed by health professionals and practitioners. Andreassen et al.’s [6] study into the participation/non participation of Roma women in a national cervical screening programme in Romania found that providers attributed low uptake to a lack of knowledge, negligence, low education and erroneous health beliefs. The Roma women by contrast, emphasised that the programme had failed to consider their needs, understandings or expectations or to the social context in which the intervention was introduced. 

Fieldwork for the current study found a similar gulf in understanding between some of the professionals interviewed and Gypsies and Travellers concerning reasons for poor health and perceptions of community health, suggesting that some professionals uncritically regurgitate what they have read in the literature even where this departs from the lived experiences of the communities that they work with. More concerning is that those with a specific remit to work with Gypsies and Travellers, and therefore with an in-depth working knowledge of these groups, were more likely to diverge from the responses of the Gypsy and Traveller participants. According to the majority of professionals in both areas for example, Gypsies and Travellers commonly experience discrimination within the healthcare system, which is contrary to the experiences reported by most Gypsy Travellers who took part in the study. Professionals in both areas mentioned the health impact of sites (poor conditions and location) and while Traveller interviewees in Ealing also mentioned this, no Gypsies in South Bucks did. Professionals in both case study areas mentioned uptake of GP registration and utilisation as a concern; however, all the Gypsies and Travellers who took part in the study were registered with and regularly use GPs, apart from those who were on the road. While many Gypsy Traveller interviewees felt that the health of their respective communities is improving, many professionals felt that the health of Gypsies and Travellers is getting worse.

Sociological accounts of health attitudes and behaviour suggest that these are shaped more by lived reality than by a coherent set of cultural values [37]. High levels of poor health among children shapes Gypsy and Travellers’ parental attitudes and practices towards their children’s health [38] to a far greater degree than the culturally grounded child-rearing practices posited by Dion [5] for example. Reluctance to engage with health services can be influenced by collective memories and experiences of historical discrimination and exclusion from health care, while a high burden of illness throughout the life course and excessive levels of premature mortality continue to influence dispositions and attitudes towards health and illness [39]. Incorporating the structural underpinnings of many supposedly Gypsy and Traveller health traits into an analysis of their health status undermines claims concerning their ‘unique beliefs about health and medical care’ [40] (p. 88). Many of the supposedly distinctive taboos and stigma surrounding conditions such as mental illness and cancer for example are shared by various social groups throughout the world [41,42]. Similarly, many of the attitudes cited as an ensemble of ‘Gypsy’ health beliefs are remarkably similar to those of other poor and marginalised populations [43,44]. 

The findings suggest that similarities in attitudes and perceptions towards health across the two case study areas represent adaptive responses to deeper structural factors some of which impact on all populations in similar socio-economic circumstances while others impact specifically on the Gypsy and Traveller population (e.g., planning laws, levels of racism and prejudice). These structural factors then manifest themselves in higher levels of poverty, poor living conditions and ill health and are mediated by a range of local level factors to produce patterned, unsynchronised and variegated outcomes in both self-reported health and subjective wellbeing in the two locations. 

## 5. Conclusions

The lack of systematic comparative studies into the health of Gypsy and Traveller communities in the UK has facilitated the perpetuation of dated and inaccurate discourses among many academics, health professionals, practitioners, and policy makers charged with improving the health of those communities [15]. Loic Wacquant’s [45] methodological agenda for a *‘comparative sociology of social polarization from below’* has as its starting point a critical evaluation of the categories and discourses employed by decision makers and social scientists that ‘under cover of describing marginality, contribute to moulding it by organising its collective perception and its political treatment’ [44] (p. 8). This critically informed approach should be combined with institutional and quantitative analysis of macro structural determinants in conjunction with ethnographic observation. The latter states Wacquant is necessary to ‘pierce the screen of discourses (that)… lock inquiry within the biased perimeter of the pre-constructed object’. From the outset he argues it is necessary to distinguish between the social conditions that characterise a locality within a hierarchical structure of places and the conditioning it entails—in this case for example, an array of the health related behaviours and strategies that develop within particular communities—and finally to specify the degree and form of involvement by state agencies and the relations that residents maintain with public officials and agencies [45] (p. 11). 

Such an approach would be a significant advance on the current state of knowledge concerning Gypsy and Traveller health and would be easier if the health authorities routinely and systematically monitored Gypsy Roma and Traveller populations as they do (albeit inconsistently) with other Black and Minority Ethnic (BME) populations. National and regional level data collected by health trusts, combined with qualitative field work and rigorous geographical comparisons could tease out the general causal dynamics from those social processes that operate at the local level, and which combine to produce diverse health outcomes between different Gypsy and Traveller populations.

## References

[1] Cromarty H. (2018). Gypsies and Travellers.

[2] Department of Communities and Local Government (DCLG) (2015). Planning Policy for Travellers Sites.

[3] Russell J., Greenhalgh T., Lewis H., MacKenzie I., Maskrey N., Montgomery J., O’Donnell J. (2013). Addressing the ‘postcode lottery’ in local resource allocation decisions: A framework for clinical commissioning groups. J. R. Soc. Med..

[4] Dion X. (2008). Gypsies and Travellers: Cultural influences on health. Community Pract..

[5] Andreassen T., Weiderpass E., Nicula F., Suteu O., Itu A., Bumbu M., Tincu A., Ursin G., Moen K. (2018). Controversies about cervical cancer screening: A qualitative study of Roma women’s (non) participation in cervical cancer screening in Romania. Soc. Sci. Med..

[6] Millan M. (2018). A Mixed Methods Comparative Case Study Exploring the Relationships between Accommodation and Planning Situations and the Experience of Health and Wellbeing for English Gypsy Communities in South Buckinghamshire and Irish Traveller Communities in Ealing. Ph.D. Thesis.

[7] Smith D., Ruston M. (2013). If you feel that nobody wants you you’ll withdraw into your own: Gypsy/Travellers, networks and healthcare utilisation. Sociol. Health Illn..

[8] Irish Traveller Movement in Britain (ITMB) (2012). Inclusion and Ethnic Monitoring of Gypsies and Travellers in the National Health Service.

[9] Parry G., Van Cleemput P., Peters J., Walters S., Thomas K., Cooper C. (2004). The Health Status of Gypsies and Travellers in England.

[10] Acton T., Caffrey S., Dunn S., Vinson P., Acton T., Munday G. (1997). Gender issues in accounts of Gypsy health and hygiene as discourses of social control. Romani Culture and Gypsy Identity.

[11] Smith D. (2018). Roma migration, anti-migrant sentiment and social integration: A case study in South-East England. Local Econ..

[12] Evans G.W., Kim P. (2010). Multiple risk exposure as a potential explanatory mechanism for the socioeconomic status-health gradient. Ann. N. Y. Acad. Sci..

[13] Williams D.R., Collins C. (1995). US socioeconomic and racial differences in health: Patterns and explanations. Annu. Rev. Sociol..

[14] Robinson D., Reeve K. (2006). Neighbourhood Experiences of New Immigration: Reflections from the Evidence Base.

[15] Department of Communities and Local Government (DCLG) (2012). Progress Report by the Ministerial Working Group on Tackling Inequalities Experienced by Gypsies and Travellers. https://assets.publishing.service.gov.uk/government/uploads/system/uploads/attachment_data/file/6287/2124046.pdf.

[16] Carr S., Lhussier M., Forster N., Goodall D., Geddes L., Pennington M., Bancroft A., Adams J., Michie S. (2014). Outreach programmes for health improvement of Traveller communities: A synthesis of evidence. Public Health Res..

[17] Spencer N., Law S., Dowler E., Spencer N. (2007). Inequalities and ethnicity: Evidence and intervention. Challenging Health Inequalities.

[18] Office for National Statistics (2017). Household Disposable Income and Inequality in the UK: Financial Year Ending 2017. https://www.ons.gov.uk/peoplepopulationandcommunity/personalandhouseholdfinances/incomeandwealth/bulletins/householddisposableincomeandinequality/financialyearending2017.

[19] Goldenberg S. (1992). Thinking Methodologically.

[20] Papworth Trust (2018). Disability in the UK 2016: Facts and Figures. http://www.papworthtrust.org.uk/sites/default/files/Disability%20Facts%20and%20Figures%202016.pdf.

[21] Mental Health Foundation (2016). Fundamental Facts about Mental Health 2016.

[22] Smith D., Greenfields M. (2012). Housed Gypsies and Travellers: Ethnicity exclusion and adaptation. Race Cl..

[23] Greenfields M., Smith D. (2010). Housed Gypsy Travellers, Social Segregation and the Reconstruction of Communities. Hous. Stud..

[24] Greenfields M., Brindley M. (2016). Impact of Insecure Accommodation and the Living Environment on Gypsies’ and Travellers’ Health.

[25] Watt P., Smets P. (2017). Social Housing and Urban Renewal: A Cross-National Perspective.

[26] Office of the Deputy Prime Minister (ODPM) (2006). The Provision and Condition of Local Authority Gypsy/Traveller Sites in England.

[27] Cemlyn S., Greenfields M., Burnett S., Matthews Z., Whitwell C. (2009). Inequalities Experienced by Gypsy and Traveller Communities: A Review.

[28] Atterbury J. (2017). Fair Access for All? Gypsies and Travellers in Sussex, GP Surgeries and Barriers to Primary Healthcare.

[29] Mc Fadden A., Siebelt L., Gayine A., Atkin K., Bell K., Innes N., Jones H., Jackson Haggi C., MacGilliyray S. (2018). Gypsy, Roma and Traveller access to and engagement with health services: A systematic review. Eur. J. Public Health.

[30] GP Bulletin (2016). What GPs Can Learn from Patient Complaints. https://www.gponline.com/gps-learn-patient-complaints/article/1419363.

[31] Scambler G. (2008). Sociology as Applied to Medicine.

[32] Lane P., Spencer S., Jones A. (2014). Experts by Experience: Reviewing UK Progress on the EU Framework for National Roma Integration Strategies.

[33] Smith D., Greenfields M. (2013). Gypsies and Travellers in Housing: The Decline of Nomadism.

[34] Joseph Rowntree Foundation (JRF) (2017). UK Poverty 2017: A Comprehensive Analysis of Poverty Trends and Figures.

[35] Atkin A., Graham H. (2009). Negotiating Ethnic Identities and Health. Understanding Health Inequalities.

[36] Scambler G. (2011). Tackling health inequalities and its pros, cons and contradictions: A commentary on Blackman, Wistow and Byrne. Soc. Sci. Med..

[37] Boxill B. (1994). The Culture of Poverty. Cult. Plur. Moral Knowl..

[38] Smith D., Newton P. (2017). Structural barriers to measles, mumps and rubella (MMR) immunisation uptake in Gypsy, Roma and Traveller communities in the United Kingdom. Crit. Public Health.

[39] Ruston M., Smith D. (2013). Gypsies/Travellers and health: Risk categorisation versus being “at risk”. Health Risk Soc..

[40] Vivian C., Dundes L. (2004). The Crossroads of Culture and Health Among the Roma (Gypsies). J. Nurs. Scholarsh..

[41] Allford A., Qureshi N., Barwel J., Lewis C., Kai J. (2014). What hinders minority ethnic access to cancer genetics services and what may help?. Eur. J. Hum. Genet..

[42] Gansler T., Henley S.J., Stein K., Nehl E.J., Smigal C., Slaughter E. (2005). Socioeconomic determinants of cancer treatment health literacy. Cancer.

[43] Quaife S.L., Winstanley K., Robb K.A., Simon A.E., Ramirez A.J., Forbes L.J.L., Brain K.E., Gavin A., Wardle J. (2015). Socioeconomic inequalities in attitudes towards cancer: An international cancer benchmarking study. Eur. J. Cancer Prev..

[44] Elkan R., Avis K., Cox K., Wilson E., Patel S., Miller S., Deepak N., Edwards C., Staniszewska S., Kai J. (2006). The reported views and experiences of cancer service users from minority ethnic groups: A critical review of the literature. Eur. J. Cancer Care.

[45] Wacquant L. (2008). Urban Outcasts: A Comparative Sociology of Advanced Marginality.

